# A Membrane-Bound NAC-Like Transcription Factor OsNTL5 Represses the Flowering in *Oryza sativa*

**DOI:** 10.3389/fpls.2018.00555

**Published:** 2018-05-03

**Authors:** Siyi Guo, Shaojun Dai, Prashant K. Singh, Hongyan Wang, Yanan Wang, Jeanie L. H. Tan, Wanyi Wee, Toshiro Ito

**Affiliations:** ^1^State Key Laboratory of Cotton Biology, Department of Biology, Institute of Plant Stress Biology, Henan University, Kaifeng, China; ^2^Temasek Life Sciences Laboratory, National University of Singapore, Singapore, Singapore; ^3^Development Center of Plant Germplasm Resources, College of Life and Environmental Sciences, Shanghai Normal University, Shanghai, China; ^4^Biological Science, Nara Institute of Science and Technology, Ikoma, Japan

**Keywords:** flowering, NAC transcription factor, rice, subcellular localization, overexpression

## Abstract

In spite of short-day (SD) nature, rice (*Oryza sativa*) shares a conserved photoperiodic network for flowering control with long-day plants like *Arabidopsis thaliana.* Flowering or heading is an important agronomic trait in rice. NAC transcription factors (TFs) are well-conserved and one of the largest families of plant TFs. However, their function in flowering or heading time is not well-known yet. A preferential expression of a membrane-bound NAC-like TF OsNTL5 in developing leaves and panicles of rice indicated to us its putative role in flowering. To examine its function, three independent constructs was generated, one with a deletion in the C terminus membrane-spanning domain (OsNTL5∆C), OsNTL5∆C fused with the SRDX transcriptional repressor motif and OsNTL5∆C used with the VP16 activation domain under the *Ubiquitin* promoter to produce the overexpressing lines *OsNTL5∆C, OsNTL5*∆C-*SRDX*, and *OsNTL5∆C-VP*, respectively in rice. The *OsNTL5*∆*C-VP* line showed an early-flowering phenotype. In contrast to this, the plants with *OsNTL5*∆C and *OsNTL5*∆C-*SRDX* showed a very strong late-flowering phenotype, suggesting that OsNTL5 suppresses flowering as a transcriptional repressor. The protein subcellular localization assay suggested that N-terminal part of the OsNTL5 is localized to the nucleus after the protein is cleaved from its membrane-spanning domain at the C-terminal end and functions as a TF. Expression of flowering genes responsible for day length signals such as *Early Heading Date 1* (*Ehd1*), *Heading Date 3a* (*Hd3a*), and *Rice Flowering Locus T1* (*RFT1*) was significantly changed in the overexpression lines of *OsNTL5*∆*C-VP, OsNTL5*∆*C*, and *OsNTL5*∆*C-SRDX* as analyzed by Quantitative Real-time PCR. ChIP-qPCR and rice protoplasts assays indicate that OsNTL5 directly binds to the promoter of *Ehd1* and negatively regulates the expression of *Ehd1*, which shows antagonistic photoperiodic expression patterns of *OsNTL5* in a 24-h SD cycle. Hence in conclusion, the NAC-like TF OsNTL5 functions as a transcriptional repressor to suppress flowering in rice as an upstream factor of *Ehd1*.

## Introduction

Flowering or “heading” is an essential process from vegetative to reproductive growth shift in plants governed by environmental and endogenous signals. Essentially, plants integrate the environmental signals (photoperiod, light quality, and temperature) as well as endogenous cues (plant developmental status) to control flowering (Simpson and Dean, 2002; Cho et al., 2016). Planting season and regional crop distribution are determined by an essential agronomic trait, heading date, or flowering time (Srikanth and Schmid, 2011; Gao et al., 2013).

Depending on the photoperiodic responses in flowering, plants can be classified into three categories: short-day (SD) plants, day-neutral plants, and long-day (LD) plants. The model plant Arabidopsis (*Arabidopsis thaliana*) is a typical LD plant, in which extended day period promotes flowering. Previous studies suggested that four genetic pathways regulate the flowering in Arabidopsis, photoperiodic pathway, autonomous pathway, gibberellins pathway, and vernalization pathway (Simpson and Dean, 2002; Boss et al., 2004; He and Amasino, 2005). According to the photoperiodic pathway, phytochromes A and cryptochromes 1 and 2 perceive light signals and the light signals are transduced to the circadian clock, which in turns influences the expression of *GIGANTEA* (*GI*) (Imaizumi and Kay, 2006). GI promotes the expression of *CONSTANS* (*CO*) in leaves, and finally this CO activates the important flowering factor, *FLOWERING LOCUS T* (*FT*) by directly binding to the promoter of *FT* (Imaizumi and Kay, 2006; Kobayashi and Weigel, 2007; Turck et al., 2008). Thus, in LD plants like Arabidopsis, flowering is promoted by the integration of circadian clock and light signals by the GI-CO-FT genetic pathway (Turck et al., 2008; Tsuji et al., 2011). Previous studies have showed that the ambient temperature is also an important factor regulating flowering in Arabidopsis. Flowering is promoted or delayed when plants are exposed to small changes of temperature. The main studies on ambient temperature focus on the genetic pathways of SHORT VEGETATIVE PHASE (SVP) and FLOWERING LOCUS M (FLM) (Lee et al., 2007, 2013; Pose et al., 2013).

In contrast to Arabidopsis, rice (*Oryza sativa*) is a classic SD plant, in which SD conditions promote heading (flowering), while LD conditions inhibit it. The molecular basis for flowering control has also been extensively studied in rice (Tsuji et al., 2011). Evolutionary studies unveil that highly homologous genes to *GI*-*CO*-*FT* photoperiodic flowering genes, *OsGI*-*Hd1* (Heading date1)-*Hd3a*, also operates in rice (Tsuji et al., 2011; Brambilla and Fornara, 2013).

The evolutionarily conserved regulatory module for photoperiodic flowering consists of GI-CO-FT signaling pathways (Hayama et al., 2003). Hd3a in rice is an ortholog of FT, the most crucial component of the GI-CO-FT flowering pathway and encodes a mobile signaling (florigen) expressed in leaves (Tamaki et al., 2007). *Hd3a* RNAi transgenic plants showed a late-flowering phenotype under SD condition, nevertheless didn’t show the late-flowering phenotype under LD conditions. This suggests the presence of another flowering regulatory factor in rice florigen, RICE FLOWERING LOCUS T 1 (RFT1) (Komiya et al., 2008). The double knock-down transgenic plants of *RFT1* and *Hd3a* were unable to flower after germination, showing that both of Hd3a and RFT1 are rice florigens (Komiya et al., 2008). Previous studies showed that OsMADS50, the homolog of SOC1 (SUPPRESSOR OF OVEREXPRESSION OF CO1/AGAMOUS-LIKE 20) was an important flowering activator controlling rice flowering (Lee et al., 2004). Recent research indicates that Ghd8 (Grain Yield, Plant Height, and Heading Date 8), NF-YCs (Nuclear Factor-YC homologs), Hd1 and PRR37 (PSEUDO-RESPONSE REGULATOR 37) regulate the expression *Hd3a* by binding with its promoter elements (Goretti et al., 2017). It has been reported that a Hd1-independent flowering pathway operates beside the conserved photoperiodic flowering pathway (GI/OsGI-CO/Hd1-FT/Hd3a/RFT). Early heading date 1 (Ehd1) regulates flowering pathway (Tsuji et al., 2011). Ehd1 belongs to a B-type response regulator, and it has been reported that it promotes flowering in the absence of *Hd1* by inducing the expression of *Hd3a* under SD conditions (Doi et al., 2004). Moreover, Ghd7 is known to inhibit the heading by regulating the expression of *Ehd1*. Meanwhile, Ghd7 also interacts and suppresses the transcriptional activity of *Hd1* (Nemoto et al., 2016; Zhang et al., 2017).

In plants, NAM, ATAF2 and CUC2 (cup-shaped cotyledon) (NAC) transcription factors (TFs) belong to one of the largest plant-specific TF families, which contains 135 and 117 NAC proteins from rice and Arabidopsis respectively (Nuruzzaman et al., 2013). Plant-specific NAC TFs have been demonstrated to be involved in various processes, such as abiotic stress resposnse, senescence, plant development, brassinosteroid signaling, and meristem regulation (Takada et al., 2001; Kim et al., 2006, 2007; Bu et al., 2008; De Clercq et al., 2013; Ng et al., 2013; Nuruzzaman et al., 2013; Xu et al., 2013; Shih et al., 2014; Chen et al., 2015). Among them, there are 8 and 5 NAC TFs with membrane-spanning domains in Arabidopsis and rice, respectively, belonging to plant MTFs (membrane-bound TFs). They are known to play important roles in plant stress response and seed germination (Seo et al., 2008; Kim et al., 2010). However, their involvement in flowering control has not been well-elucidated.

The present study employed a reverse genetic approach to unveil the functions of a membrane-bound NAC transcription factor (OsNTL5) from *Oryza sativa*. Our results suggest that OsNTL5 is translocated from ER (endoplasmic reticulum) to nucleus and acts as a repressor of rice flowering by directly regulating the expression of *Ehd1*.

## Materials and Methods

### Plant Materials and Growth Conditions

Nipponbare, a japonica rice (*Oryza sativa*) cultivar, was used in this work. Nipponbare and transgenic plants were grown in the greenhouse at 30–32°C for 12.5 h (light) and 24–26°C (dark) in Singapore.

### Vector Construction and Plant Transformation

The *OsNTL5* cDNA that lacks the C-terminal membrane spanning domain (amino acid 1–547, cDNA 1–1641 bp) was amplified by RT-PCR (using primers shown in Supplementary Table 1) and then inserted into pUN1301 driven by the maize *Ubiquitin* promoter (*OsNTL5∆C*). To produce *OsNTL5∆C-SRDX*, the DNA sequence encoding SRDX peptide (LDLDLELRLGFA) was involved in the reverse primer, which contains a stop codon (TGA) at the end of the SRDX coding sequences, and the *OsNTL5∆C-SRDX* PCR product was inserted into the pUN1301 vector. To obtain *OsNTL5∆C-VP*, the DNA sequences encoding VP16 peptide (DALDDFDLDML) and stop codon were included in the reverse primer, and the *OsNTL5∆C-VP* PCR product was inserted into the pUN1301 vector. These three constructs were fused with HA-tag in the N terminus. The constructs were transferred to *Agrobacterium tumefaciens* strain AGL1 and then transformed into rice with the *Agrobacterium*-mediated method. PCR products were confirmed by sequencing and sequences of all primers are listed in Supplementary Table 1.

### Subcellular Localization Assay

The full-length and truncated coding sequences of *OsNTL5, OsNTL5∆C*, and *OsNTL5TM* (transmembrane domain alone) were PCR-amplified from rice cDNA, and then generated *35S::GFP-OsNTL5, 35S::GFP-OsNTL5∆C*, and *35S::OsNTL5TM-GFP*. The nucleic marker *35S::H2B-mCherry* (Rosa et al., 2014) and the ER marker ER-rk (Nelson et al., 2007) were transiently co-transformed together with *35S::GFP-OsNTL5, 35S::GFP-OsNTL5∆C* or *35S::OsNTL5TM-GFP* and ER-rk in rice protoplasts by polyethylene glycol (PEG)-mediated transformation method as previously described (Guo et al., 2013). The transformed protoplasts were examined by the Zeiss confocal microscopy (LSM710).

### Quantitative RT-PCR

Total RNA was extracted from 14-day-old WT and transgenic plants for *NTL5∆C, NTL5∆C-SRDX*, and *NTL5∆C-VP* using the RNeasy Plant Mini Kit (QIAGEN, Germany). Two μg total RNA was used to synthesize the first-strand cDNA using SuperScript^TM^ III Reverse Transcriptase (Invitrogen). For quantitative RT-PCR in rice, the rice ubiquitin (Ubq) was used as an internal control. We performed the expression analysis of *Ehd1, Hd3a, RFT, MADS50, Ghd7*, and *OsGI* in WT under the SD condition according to the method reported previously (Doi et al., 2004). Whereas, for qRT-PCR in rice protoplasts, the expression of *Ehd1* and *OsNTL5* was assayed as the *luciferase* gene as a control. The specific primer pair for the *GFP* gene was used for the detection of the expression of *Ehd1pro::GFP* and *Ehd1pro∆E::GFP.* All the qRT-PCR assays were done in triplicate. The relative transcript level of a gene of interest is calculated as 2^-∆∆CT^ [∆∆C_T_ = ∆C_T_ (candidated sample) – ∆C_T_ (control sample); ∆C_T_ = C_T_ (interesting gene) – C_T_ (internal control gene)]. The qRT-PCR assays were done in triplicate. The primers for qRT-PCR experiments were listed in Supplementary Table 1.

### Phylogenetic Analyses

The phylogenetic analysis of 7 and 26 NAC protein sequences from Arabidopsis and rice was performed using MEGA 7, with default settings and the neighbor-joining tree (NJT) method. Bootstrap values (%) of 1,000 replicates are shown at the branching points.

### ChIP Assays

For the ChIP assay, 14-day-old seedlings of WT and *OsNTL5∆CSRDXOE10* were used as materials. The detailed ChIP experiment was done as described previously (Gan et al., 2014). Total chromatin was immunoprecipitated using with anti-HA (Santa Cruz Biotechnology, #sc-7392). The mouse IgG (Santa Cruz Biotechnology, #sc-2025) was used as a control ChIP assay. DNA fragments were collected using PCR purification Kit. ChIP-qPCR was performed on the ABI PRISM 7900HT real-time PCR machine (Applied Biosystems) with the KAPA SYBR FAST ABI Prism qPCR Master Mix (KAPA Biosystems). The promoter of *UBI* (Os01g45400) was used as a negative control. The ChIP relative enrichment of HA-NTL5∆CSRDX10 binding was calculated from the ratio between anti-HA antibody and normal mouse IgG immunoprecipitated DNA (Gan et al., 2014). The ChIP-qPCR assays were done in triplicate. Primers for the ChIP assays are listed in Supplementary Table 1.

### *In Situ* Hybridization

Seven-day-old leaves and young inflorescences were fixed in 4% paraformaldehyde at 4°C overnight. The materials were then dehydrated through an ethanol series and histoclear, embedded into paraffin, sectioned to 7 μm. For the synthesis of the *OsNTL5* antisense and sense probe, Digoxigenin-labeled hybrid probes were transcribed *in vitro* from cDNA of *OsNTL5* with specific primers (Supplementary Table 1) (Guo et al., 2013). The results were photographed under a Zeiss microscope.

### Western Blotting

Total proteins from 10-day-old WT and *OsNTL5∆C-SRDX10* transgenic rice expressing HA-*OsNTL5∆C-SRDX* were extracted using 2× SDS-PAGE gel-loading buffer, heat-denatured, and separated in an SDS-PAGE gel. Immunoblotting detected HA-OsNTL5∆CSRDX with anti-HA (Santa Cruz Biotechnology, #sc-7392).

## Results

### OsNTL5 Suppresses Flowering in Rice

**FIGURE 1:**
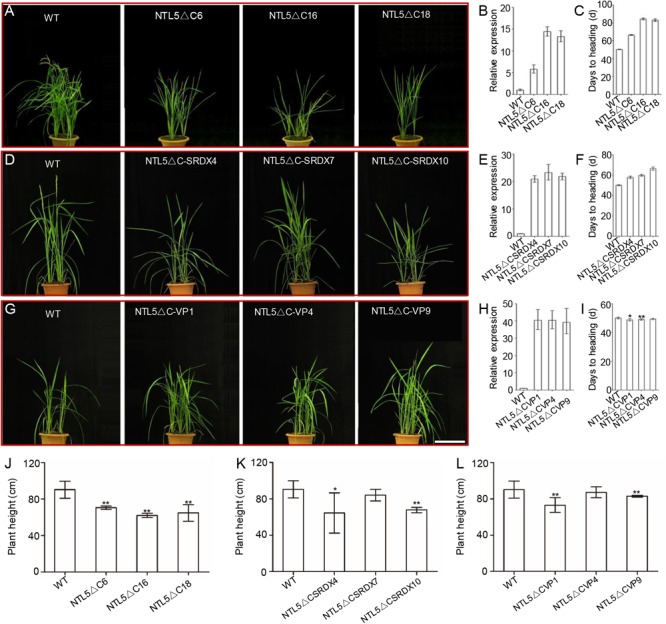
Phenotypic and molecular characterization of the *OsNTL5∆C, OsNTL5∆C-SRDX*, and *OsNTL5∆C-VP* transgenic lines of rice. **(A)** The phenotypes of 60-day-old WT and *OsNTL5∆C* transgenic plants. Bar = 20 cm. **(B)** Relative expression of *OsNTL5* in the *OsNTL5∆C* transgenic lines. Values represent the means ± SD. **(C)** Days of heading to WT and *OsNTL5∆C* under natural photoperiodic conditions. Values represent the means ± SD (standard deviation). The data were collected from 20 individual plants. **(D)** Phenotypes of 55-day-old WT and *OsNTL5∆C-SRDX* transgenic plants. Bar = 20 cm. **(E)** Relative expression of *OsNTL5* in the *OsNTL5∆C-SRDX* transgenic lines. Values represent the means ± SD. **(F)** Days of heading to WT and *OsNTL5∆C-SRDX* under natural photoperiodic conditions. Values represent the means ± SD. The data were collected from 20 individual plants. **(G)** Phenotypes of 55-day-old WT and *OsNTL5∆C-VP* transgenic plants. Bar = 20 cm. **(H)** Relative expression of *OsNTL5* in the *OsNTL5∆C-VP* transgenic lines. Values represent the means ± SD. **(I)** Days of heading to WT and *OsNTL5∆C-VP* under natural photoperiodic conditions. Values represent the means ± SD. Data were collected from 20 individual plants. **(J)** Plant height of WT and *OsNTL5∆C* was measured 85 days after germination. Values represent the means ± SD, *n* = 15. **(K)** Plant height of WT and *OsNTL5∆C-SRDX* was measured 85 days after germination. Values represent the means ± SD, *n* = 15. **(L)** Plant height of WT and *OsNTL5∆C-VP* was measured 85 days after germination. Values represent the means ± SD, *n* = 15. ^∗^*P* < 0.05, ^∗∗^*P* < 0.01.

A phylogeny was constructed by using 26 and 7 protein sequences of membrane-bound NAC transcription factors (MTFs) from rice and Arabidopsis, respectively to explore the evolutionary relationships of OsNTL5 of rice with other MTFs in rice and Arabidopsis. The phylogeny result demonstrated that OsNTL5 (Os08g44820) is a rice orthologous to Arabidopsis ANTHER INDEHISCENCE FACTOR (AIF) with 60% identity and there is another homolog of OsNTL6 (Os02g57650), which may share at least a partial redundancy with OsNTL5 (Supplementary Figure 1). Conserved domains, i.e., NAC and NAC repression domain (Seo et al., 2008; Shih et al., 2014) of OsNTL5 showed similarity with AIF, NST1 (NAC SECONDARY WALL THICKENING PROMOTING FACTOR1) and NST2 (Supplementary Figures 2A,B). To explore the function of OsNTL5, three kinds of independent overexpressing transgenic rice lines, i.e., *OsNTL5∆C, OsNTL5∆C-SRDX*, and *OsNTL5∆C-VP* were produced by using constructs *pUbi::HA:OsNTL5∆C, pUbi::HA:OsNTL5∆C-SRDX*, and *pUbi::HA:OsNTL5∆C-VP16* (**Figure 1**). More than 10 independent transgenic lines were obtained for each construct. All independent transgenic lines of *OsNTL5∆C* showed late flowering, shorter seedlings, reduced plant height, and reduced fertility phenotypes (**Figures 1A,C,J** and Supplementary Figures 3A, 4A) and *OsNTL5∆C-SRDX* transgenic rice, demonstrated similar phenotypes (**Figures 1D,F,K** and Supplementary Figures 3B, 4B). In contrast, the *OsNTL5∆C-VP* transgenic plants (*OsNTL5∆C-VP1* and *OsNTL5∆C-VP4*) showed early flowering, shorter seedlings reduced plant height, and sterile phenotypes (**Figures 1G,I,L** and Supplementary Figures 3C, 4C). The qRT-PCR data demonstrated that the transcripts of *OsNTL5* transgene were significantly enhanced in all the transgenic lines, i.e., *OsNTL5∆C, OsNTL5∆C-SRDX*, and *OsNTL5∆C-VP* (**Figures 1B,E,H**). Hence, the results of phenotypic analysis of these transgenic lines confirm that OsNTL5 regulates flowering in rice as a transcriptional repressor.

### Cleavage of the Transmembrane Domain of OsNTL5 Targets Nucleus

**FIGURE 2:**
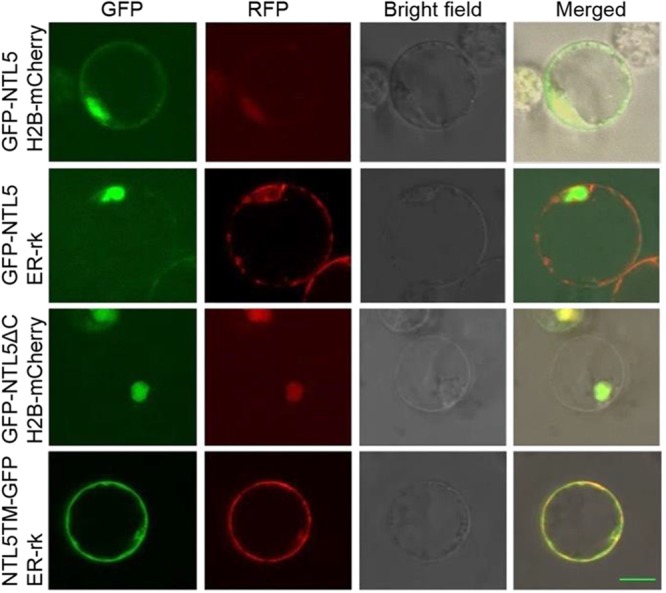
Subcellular localization of OsNTL5, OsNTL5∆C, and OsNTL5TM in protoplasts of rice. Confocal images of cells expressing GFP-OsNTL5, GFP-OsNTL5∆C, and NTL5TM-GFP. Nuclear marker protein (H2B-mCherry) and ER marker protein (ER-rk) were co-expressed respectively in rice leaf protoplasts by PEG (polyethylene glycol) treatment. Fluorescence was observed using a Zeiss LSM 710 confocal microscope. TM, transmembrane domain. Bar = 10 μm.

Membrane spanning domain containing NAC transcription factor NTL family TFs are released from ER or membrane for modulation of expression of target genes to the nucleus (Ng et al., 2013; Nuruzzaman et al., 2013; Shih et al., 2014). To verify that OsNTL5 of rice is also modulated, the GFP tag was fused as a N-terminal fusion with the full length *OsNTL5* cDNA (GFP-OsNTL5), *OsNTL5* cDNA lacking the C-terminal end of its protein (GFP-OsNTL5∆C) and the *OsNTL5* cDNA which encodes only a transmembrane domain (OsNTL5TM-GFP) and transformed into the protoplasts of rice (**Figure 2**). The results of subcellular localization demonstrated that OsNTL5 was localized to both nuclei as well as ER, while OsNTL5∆C was expressed only in the nucleus and OsNTL5TM colocalized with the ER marker (**Figure 2**). Thus, the subcellular localization study suggests that the TM domain is responsible for targeting to ER and the cleavage of the TM domain leads to nucleus localization.

### OsNTL5 Is Expressed Preferentially in Leaves and Inflorescences

**FIGURE 3:**
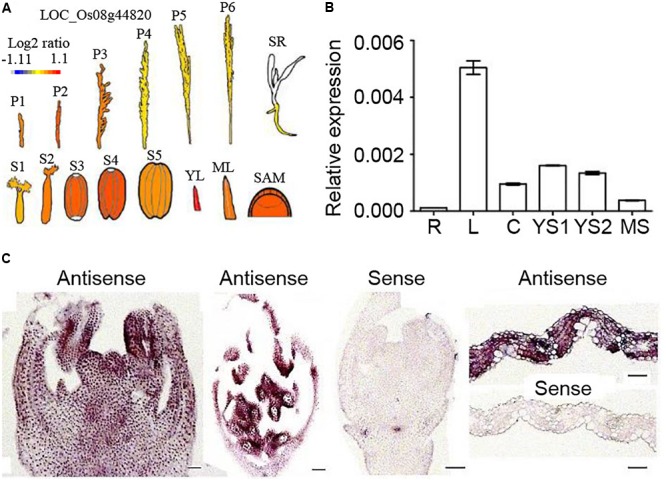
Expression patterns of *OsNTL5*. **(A)** Relative expression levels of *OsNTL5* in inflorescences, seedlings, seeds, young leaves, mature leaves and SAM based on microarray data showed in the eFP browser (http://www.bar.utoronto.ca/efp/cgi-bin/efpWeb.cgi). The color scale shows microarray signal level. P1, Young Inflorescence (P1); P2, Inflorescence (P2); P3, Inflorescence (P3); P4, Inflorescence (P4); P5, Inflorescence (P5); P6, Inflorescence (P6); SR, Seedling Root; S1-5, Seed S1-5; YL, Young Leaf; ML, Mature Leaf; SAM, Shoot Apical Meristem. **(B)** Relative expression of *OsNTL5* in roots (R), leaves (L), culms (C), young inflorescence stages1 (YS1 and YS2), Mature inflorescence stage by real-time PCR. **(C)** RNA *in situ* hybridization of *OsNTL5* using anti-sense and sense probes specific to *OsNTL5* in inflorescence, mature flower, and leaf. Bars = 50 μm.

The eFP bar browser was searched for the *OsNTL5* expression profile, and the result of this search in **Figure 3A** revealed that *OsNTL5* was highly expressed in young leaves, SAM (shoot apical meristem), seed developmental stages (S1 to S4) and young inflorescences (P1 to P3). The qRT-PCR results confirmed that *OsNTL5* was highly expressed in young leaves, immature stages of inflorescence, culms and mature stages inflorescences, and weakly in roots (**Figure 3B**). Furthermore, *in situ* hybridization experiment was done in various tissues. An high levels of *OsNTL5* transcript was detected in inflorescences, anthers, pollens, and leaves, while the sense probe showed no such signals in these tissues (**Figures 3C,D**). Together with eFP browser data and transcript results suggest that *OsNTL5* is expressed in leaves and inflorescences.

### OsNTL5 Is a Negative Regulator of *Ehd1*

**FIGURE 4:**
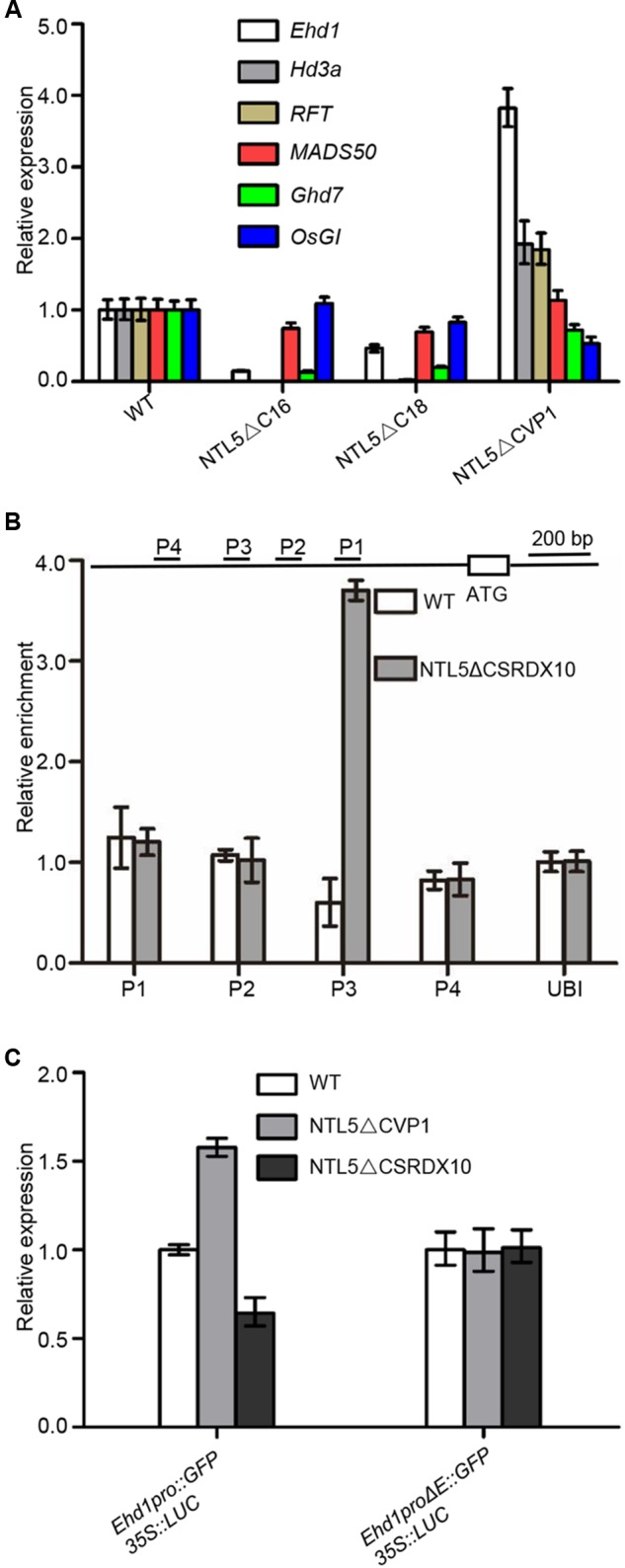
OsNTL5 negatively regulates the *Ehd1* transcript. **(A)** Expression analyses of *Ehd1, Hd3, RFT, MADS50, Ghd7*, and *OsGI* transcripts in *OsNTL5∆C* transgenic line 16#, 18# and *OsNTL5∆C-VP* transgenic line 1# by qRT-PCR. *Ubiquitin* (Os03g13170) was used as an internal control. The qRT-PCR experiments were performed in three biological replicates. The data were means ± SD (standard deviation). **(B)** ChIP binding assay of OsNTL5 with the promoter of *Ehd1*. Immunoprecipitation (IP) was pulled down by the anti-HA antibody in *NTL5∆C-SRDX10* transgenic plants. In control, immunoglobulin G was used to precipitate the same plant materials and the value of PCR was set as 1. **(C)** Effect of OsNTL5 on transcriptional regulation of *Ehd1* in rice protoplasts. *Ehd1pro∆E* stands for deletion of the one *cis*-element (CTCCACGT) on the promoter of *Ehd1* (at -1490 upstream from the start codon at the primer set P3). *35S::Luciferase* was as an internal control. The OsNTL5 on transcriptional regulation of *Ehd1* experiments were performed in three biological replicates. The data were means ± SD (standard deviation).

Next in order to analyze the genetic hierarchy of OsNTL5 in rice flowering pathways, we analyzed the expression of the flowering marker genes; *Ehd1, Hd3a, RFT1, MADS50, Ghd7, OsGI, Hd1, Ehd2, Ehd3, Ehd4*, and *DTH8* in *OsNTL5∆C* (lines #16 and #18) and *OsNTL5∆C-VP* (line #1). Compared to WT, *Ehd1, Hd3a*, and *RFT1* showed siginificantly reduced expression levels in *OsNTL5∆C*, while they are increased (∼4 fold for *Ehd1* and ∼2 fold for *Hd3a* and *RFT1*) in *OsNTL5∆C-VP* (**Figure 4A**). Meanwhile, *OsMADS50* and *OsGI* did not show significant changes (**Figure 4A**). Expression of *Ghd7, Hd1, Ehd2, Ehd3, Ehd4*, and *DTH8* were also altered in these transgenic lines (Supplementary Figure 5).

**FIGURE 5:**
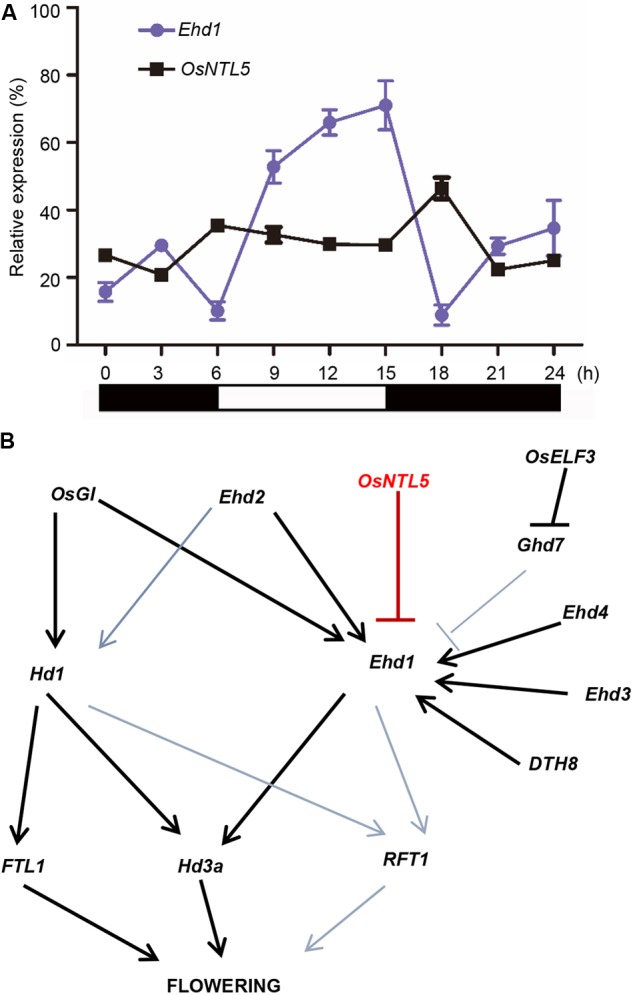
Photoperiodic expression analysis of OsNTL5 and a working model of flowering regulation based on our findings in rice. **(A)** *Ehd1* and *OsNTL5* mRNA levels in WT (Nipponbare) under short day (SD) conditions. The black and white bars at the bottom represent the dark and light periods, respectively. The qRT-PCR experiments were performed in three biological replicates. The data were means ± SD (standard deviation) **(B)** Working model in rice flowering pathway of OsNTL5 as a modulator of *Ehd1* expression and flowering repressor.

Among the genes examined, *Ehd1* showed the most obvious upregulation in the dominant active line (*OsNTL5-VP*) and reduced expression in the overexpression line (*OsNTL5∆C*). Further, *Ehd1* functions as a hub gene in the flowering pathway (**Figure 5B**). Therefore, we tested whether OsNTL5 acted through *Ehd1*. We preformed Western blotting to check the expression of *OsNTL5∆CSRDX10* using anti-HA antibody (Supplementary Figure 6). ChIP-qPCR (chromatin immunoprecipitation) analysis performed to determine whether OsNTL5 directly binds to *Ehd1* or not using anti-HA antibody in *OsNTL5∆CSRDX* line #10 transgenic plants. We detected the specific binding of the OsNTL5 protein to the upstream region of *Ehd1* (primer set P3) about 1400 bp from the transcription start site (**Figure 4B**).

Furthermore, to understand the effect of OsNTL5 on the transcriptional activity of *Ehd1* in *OsNTL5∆C-VP1* and *OsNTL5∆C-SRDX10* lines, we performed a transient expression assay in rice protoplasts produced from WT, *OsNTL5∆C-VP1*, and *OsNTL5∆C-SRDX10* plants. Previous studies showed that NAC transcription factors bind with the 4-bp conserved *cis*-element (CACG) (Tran et al., 2004; Sakuraba et al., 2015). Bioinformatics analysis indicated that there were one *cis*-elements (CACG) around the binding site of OsNTL5 (primer set P3; 1.4 kb upstream region from the transcription start site of *Ehd1*. We deleted the *cis-*elements (E, CTC**CACG**T) and produced *Ehd1∆E::GFP.* Next, we transformed the protoplasts with *Ehd1pro::GFP* and *Ehd1∆E::GFP*, by using *35S::Luciferase* as a control. The expression of *Ehd1pro::GFP* was significantly enhanced in *OsNTL5∆C-VP* protoplasts, while contrary to this a decreased in *OsNTL5∆C-SRDX* protoplasts compared to WT. However, the expression of *Ehd1∆E::GFP* was not altered in these assays (**Figure 4C**). These results clearly showed that OsNTL5 directly binds to the *Ehd1* promoter and represses its expression.

Since *Ehd1* shows circadian oscillation, we tested the expressional correlation between *OsNTL5* and *Ehd1*. The qPCR data showed that *OsNTL5* possess a diurnal expression contrary to the expression pattern of *Ehd1* at the same time point in seedlings grown under the SD (**Figure 5A**). These results suggest that OsNTL5 can directly regulate the expression of *Ehd1* to influence photoperiodic flowering pathway in rice.

## Discussion

In spite of SD nature, rice (*Oryza sativa*) shares a conserved photoperiodic genetic network for flowering with typical LD plants like *Arabidopsis thaliana*. NAC transcription factors are highly conserved and known to regulate developmental and environmental responses (Chen et al., 2008; Kim et al., 2010). Arabidopsis and rice are known to have 18 and 5 membrane-bound NAC transcription factors (MTFs; designated as NTLs). Previous studies showed that NTLs proteins localized to the nuclear and plasma membrane, the nuclear and ER, was cleaved by some factors and moved from plasma membrane or ER to the nucleus (Kim et al., 2006; Chen et al., 2008; De Clercq et al., 2013; Ng et al., 2013). Our subcellular localization data demonstrated that OsNTL5 targeted to the nuclear and ER, we also hypothesize that OsNTL5 may be cleaved by some unknown factors and release from ER to nucleus (**Figure 2**). An enhanced accumulation of OsNTLs under abiotic stress environment identified them as a critical component of stress-responsive gene regulatory network in rice (Kim et al., 2010). However, in rice their function in developmental process including flowering control has not been elucidated. Here, we first identify that OsNTL5 controls flowering or heading through the direct repression of the photoperiodic flowering gene *Ehd1*. The results of ChIP-qPCR and luciferase assays proved our hypothesis that on binding with the conserved *cis*-element present in the promoter region of *Ehd1*, OsNTL5 regulated its expression (**Figure 4B**). Furthermore, this binding leads to a reduced expression of *Ehd1* in transgenic plants compared to wild-type, confirmed by qRT-PCR results (**Figure 4C**). *Ehd1* has been reported to show a diurnal expression pattern under the SD condition (Doi et al., 2004). Accordingly, our study demonstrated that *OsNTL5* and *Ehd1* possess antagonistic diurnal expression patterns at the same time point (**Figure 5A**). Our findings show that OsNTL5 is a key modulator that regulates the flowering in rice by binding with the promoter region of *Ehd1* and controlling its expression under SD condition (**Figure 5B**).

Out of the five Membrane-bound NAC transcription factor family (OsNTL2-6) of rice surprisingly, we found that OsNTL5 and OsNTL6 show high similarity to Arabidopsis Anther Indehiscence Factor (AIF), which is known to regulate the anther dehiscence by controlling the expression of JA biosynthesis pathway genes in Arabidopsis (Shih et al., 2014). However, *AIF* and *OsNTL5* have different expression profile across the different tissues from Arabidopsis and rice respectively (Kim et al., 2010; Shih et al., 2014). Hence, it can be hypothesized that OsNTL5 has a divergent function with Arabidopsis AIF. Phylogenetic analysis has shown that OsNTL5 and OsNTL6 have high similarity in protein sequences, meanwhile, *OsNTL6* is also expressed in a similar pattern with *OsNTL5* through eFP bar bioinformatics data (**Figure 3A** and Supplementary Figure 7), these data indicate that OsNTL5 and OsNTL6 have functional redundancy. In fact, We have obtained the mutant seeds of OsNTL5 from RiceGE (Accession No.: PFG_3A-16506.L in Rice Functional Genomic Express Database), but we didn’t observe any obvious flowering defects in the OsNTL5 mutant line when compared to wild-type.

In the flowering network of SD plant rice, the OsGI-Hd1-Hd3a pathway promotes the flowering especially in SD conditions, whereas Ehd1 is also reported to enhance rice flowering by inducing the expression of *Hd3a* (Chen et al., 2008). The expression of *Hd3a* is also regulated by other factors like Ehd2 and OsMADS51 besides blue light (Chen et al., 2008). Interestingly, the results of our qPCR demonstrated an altered expression pattern of flowering marker genes like *Hd3a, RFT1, OsMADS50, Ghd7, Hd1, Ehd2, Ehd3, Ehd4*, and *DTH8* in *NTL5∆C* and *NTL5∆C-VP16* transgenic plants under SD conditions. These results indicate that OsNTL5 directly and/or indirectly regulates multiple flowering genes in rice (**Figure 4A** and Supplementary Figure 5). Hence, in conclusion, our study demonstrated that the OsNTL5 of *Oryza sativa* is an ortholog of Arabidopsis AIF, which modulate the expression of *Ehd1* by binding with its regulatory element and delays the onset of flowering by migrating from ER to nucleus. However, the factors involved in the cleavage of OsNTL5 from ER remain to be elusive. Thus the result of our study is a valuable addition to the on-going studies on improvement in rice production.

## Accession Numbers

Sequence data used in this article can be found in the NCBI database under the following Accession Nos.: Os08g44820 (OsNTL5) and OsNTL6 (Os02g57650).

## Author Contributions

TI and SG conceived and directed the project. SG designed, performed all experiments and chemical analysis, and the integrated data analysis. SG, SD, HW, YW, JT, WW, and PS interpreted the data and wrote the manuscript with the assistance and approval of all authors.

## Conflict of Interest Statement

The authors declare that the research was conducted in the absence of any commercial or financial relationships that could be construed as a potential conflict of interest.
