# Supplementary material for: A Membrane-Bound NAC-Like Transcription Factor OsNTL5 Represses the Flowering in Oryza sativa
Source: Front Plant Sci. 2018 May 3;9:555. doi: 10.3389/fpls.2018.00555 (PMC5943572; doi:10.3389/fpls.2018.00555)
Supplement: Supplementary file 1 [file Table_1.pdf]

Supplemental Table 1 │ Primers used in this study.

Experiment	Primer	Sequence (5’-3’)
Real-time PCR	NTL5-qRT-F	ACCATTGCCTTGCCAGTTTCTGAC
	NTL5-qRT-R	CCACAGGCTCAACATGGTGAAGAA
	ACT-qRT-F	TCTTACGGAGGCTCCACTTAAC
	ACT-qRT-R	TCCACTAGCATAGAGGGAAAGC
	Ubq-qRT-F	AACCAGCTGAGGCCCAAGA
	Ubq-qRT-R	ACGATTGATTTAACCAGTCCATGA
	Ehd1-qRT-F	GGATGCAAGGAAATCATGGA
	Ehd1-qRT-R	AATCCCATCGGAAATCTTGG
	Hd3a-qRT-F	GCTCACTATCATCATCCAGCATG
	Hd3a-qRT-R	CCTTGCTCAGCTATTTAATTGCATAA
	RFT1-qRT-F	TGGGTTAGCTGACCTAGATTCAAA
	RFT1-qRT-R	GCCAACCACAAGAGGATCGT
	OsMADS50-qRT-F	CAGGCCAGGAATAAGCTGGAT
	OsMADS50-qRT-R	TTAGGATGGTTTGGTGTCATTGC
	Ghd7-qRT-F	AGGTGCTACGAGAAGCAAATCC
	Ghd7-qRT-R	GGGCCTCATCTCGGCATAG
	OsGI-qRT-F	GATGGTGTGGTGGAGTCATG
	OsGI-qRT-R	TTGTTGGAGGCTTCAATTCTC
	GFP-qRT-F	TCAAGATCCGCCACAACATC
	GFP-qRT-R	GTGCTCAGGTAGTGGTTGTC
	LUC-qRT-F	GTCCTATGATTATGTCCGGTTATGT
	LUC-qRT-R	GTCTTCGTCCCAGTAAGCTATG
ChIP-qPCR	ChIP-P1F	CTCATGCATGGAGGTGATTATTTC
	ChIP-P1R	TTAAGCAGCTATATGTGCAAAGC
	ChIP-P2F	CTAGCTAGCTGGAGGAGGAA
	ChIP-P2R	GGTCAATTTCACCTAATTCAGTGTT

	ChIP-P3F	ATTGCGGTTGTGGAGGAA
	ChIP-P3R	AGGTAGCTAGAGATCTAACTCAGG
	ChIP-P4F	GCGCAATCGCATACACAATAA
	ChIP-P4R	GTGGTGCAAATATGCAAGATCAA
	ChIP-UBIF	GGACTGGTTAAATCAATCGTCA
	ChIP-UBIR	CCATATACCACGACCGTCAAAA
Transcription activity analysis	Ehd1proGaF	GGGGACAAGTTTGTACAAAAAAGCAGGCTGCCCTACACGTCGCTAATCTACAC
	Ehd1proGaR	GGGGACCACTTTGTACAAGAAAGCTGGGTGCAGCTCTCGGTGATCCATTATT
	Ehd1proΔEF	GGAGGAAGGGAGCTCGCCATGGCCGT
	Ehd1proΔER	ACGGCCATGGCGAGCTCCCTTCCTCC
Subcellular localization	NTL5TMGaF	GGGGACAAGTTTGTACAAAAAAGCAGGCTGCATGGCAGTACTGCGTGGTGGCTTC
	NTL5ΔCNGaR	GGGGACCACTTTGTACAAGAAAGCTGGGTGGGTTATTGTGTTGCAGCCAAAG
	NTL5GaF	GGGGACAAGTTTGTACAAAAAAGCAGGCTGCATGAGCCACCCCTCGTCG
	NTL5GaF	GGGGACCACTTTGTACAAGAAAGCTGGGTGCTACTTGCCATAGATGCACATGC
	NTL5GaR	GGGGACCACTTTGTACAAGAAAGCTGGGTGCTACTTGCCATAGATGCACATGC

NTL5TMGaF	GGGGACAAGTTTGTACAAAAAAGCAGGCTGCAT GGCTCCCCCTGCTTTTGC
NTL5TMGaR	GGGGACCACTTTGTACAAGAAAGCTGGGTGCTT GCCATAGATGCACATGCC
