# Supplementary material for: A Membrane-Bound NAC-Like Transcription Factor OsNTL5 Represses the Flowering in Oryza sativa
Source: Front Plant Sci. 2018 May 3;9:555. doi: 10.3389/fpls.2018.00555 (PMC5943572; doi:10.3389/fpls.2018.00555)
Supplement: Supplementary file 2 [file Data_Sheet_1.PDF]

Supplemental Figure 1

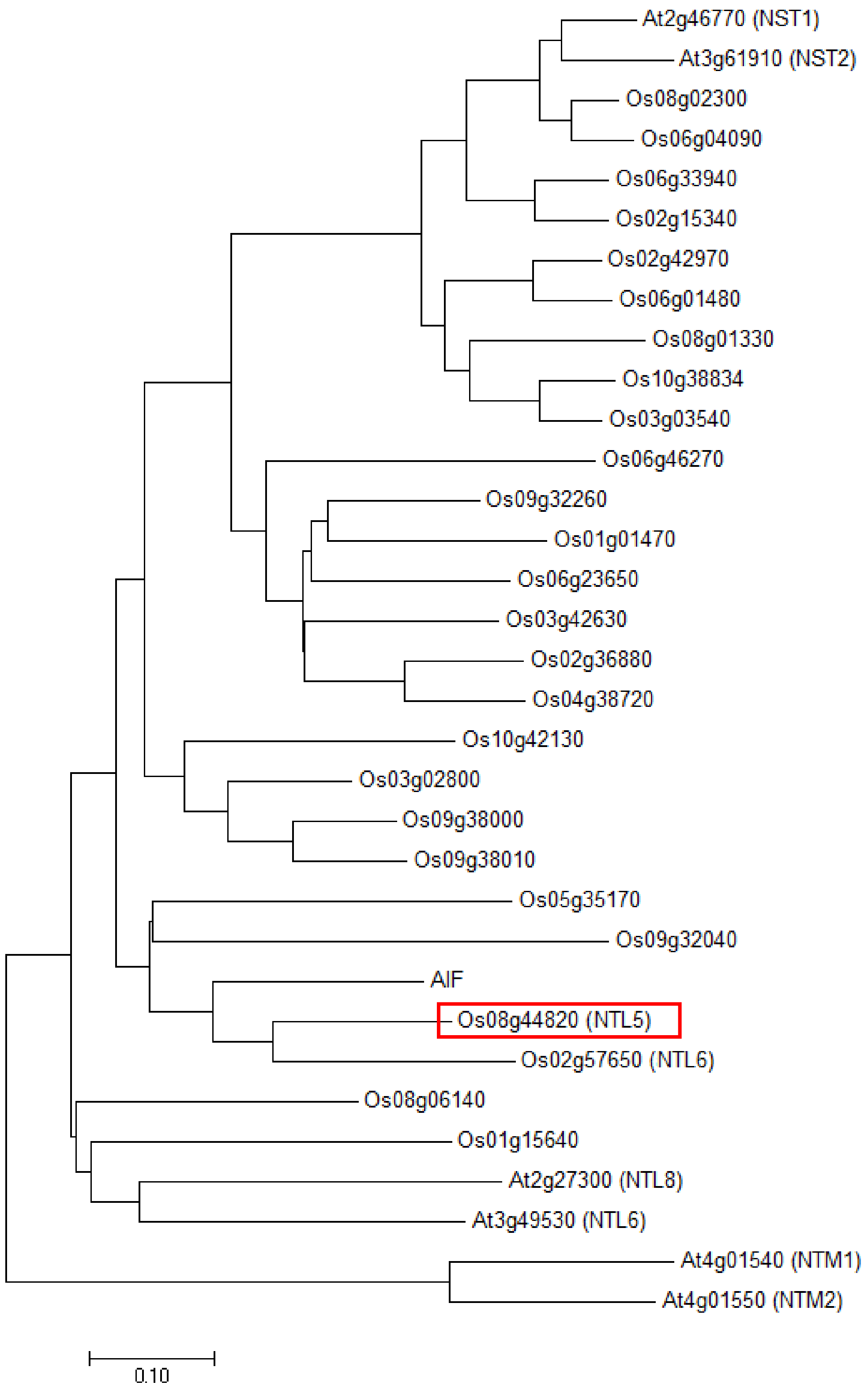

Supplemental Figure 1│ Phylogenetic tree analysis of 26 rice and 7 *Arabidopsis* NAC-like transcription factors (NTLs).

Supplemental Figure 2

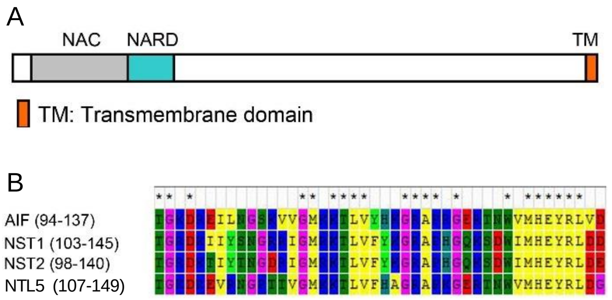

Supplemental Figure 2│ Conserved domain architecture of OsNTL5. (A) The OsNTL5 protein contains a conserved NAC domain, NARD domain, a variable transmembrane (TM) domain. **(B)** OsNTL5 includes a conserved NARD domain for the repression of transcriptional activation involved in several other NAC- like proteins (AIF, NST1, and NST2).

Supplemental Figure 3

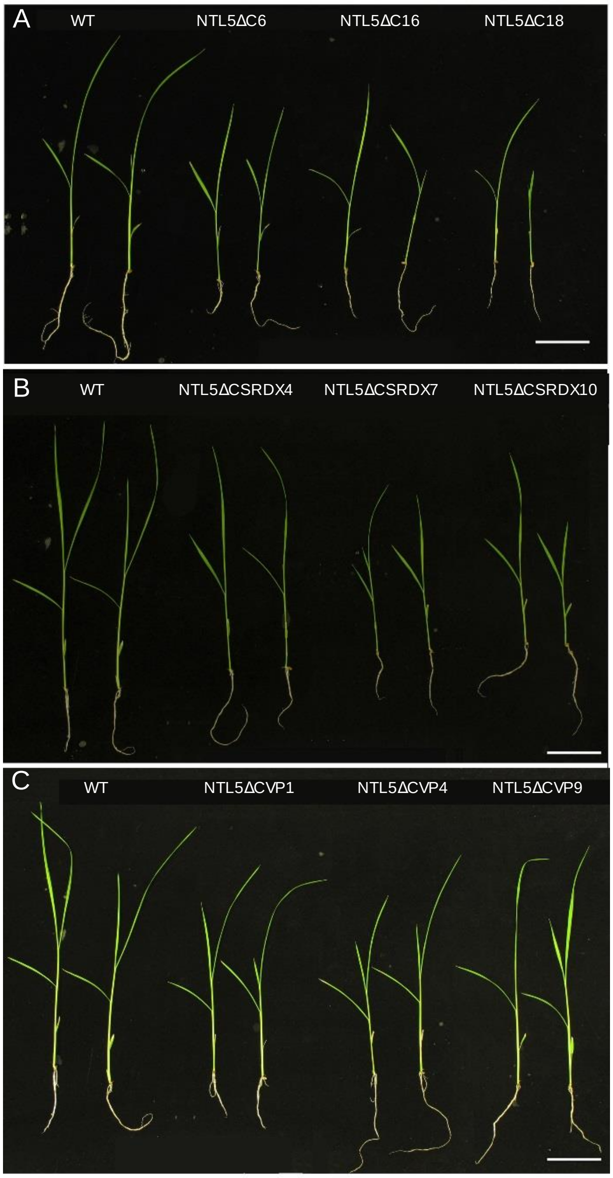

Supplemental Figure 3│The phenotype of *OsNTL5ΔC* (A), *OsNTL5ΔC-SRDX* (B), *OsNTL5ΔC-VP* transgenic plants in the 10-day-old seedling stage (C). Bars = 5 cm.

Supplemental Figure 4

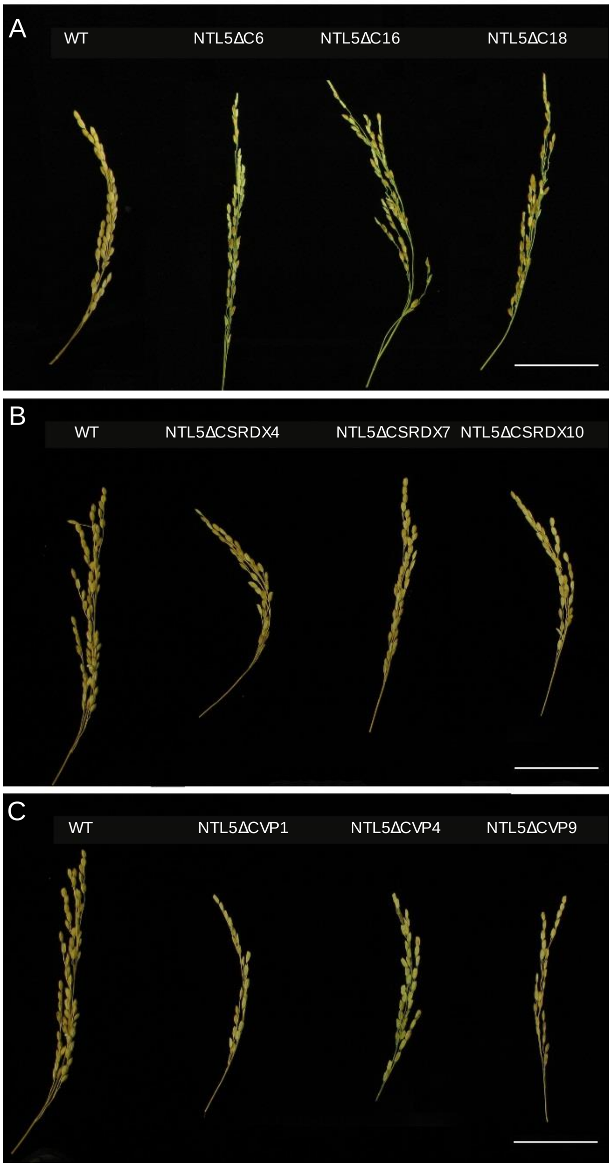

Supplemental Figure 4│ Panicles phenotype of *OsNTL5ΔC*, *OsNTL5ΔC-SRDX*, *OsNTL5ΔC-VPs* transgenic plants. Bars = 5 cm.

Supplemental Figure 5

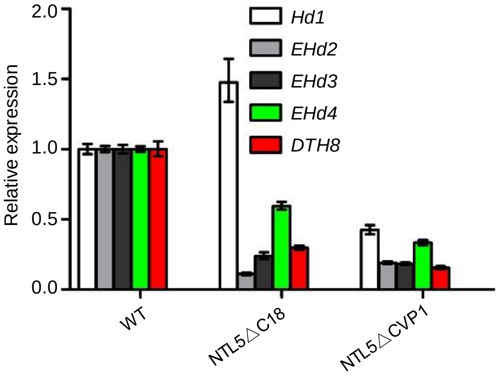

Supplemental Figure 5│ qRT-PCR analysis of *Hd1*, *Ehd2*, *Ehd3*, *Ehd4*, and *DTH8* in *OsNTL5ΔC18* and *OsNTL5ΔC-VP1#* transgenic lines. Standard deviation is based on two biological and three technical replicates.

Supplemental Figure 6

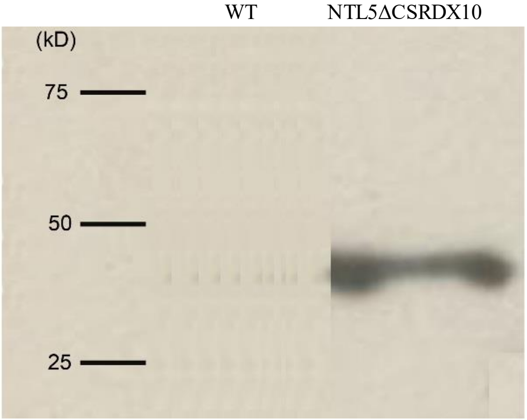

Supplemental Figure 6 | Western blot analysis of *OsNTL5ΔC-SRDX10* transgenic rice.

Supplemental Figure 7

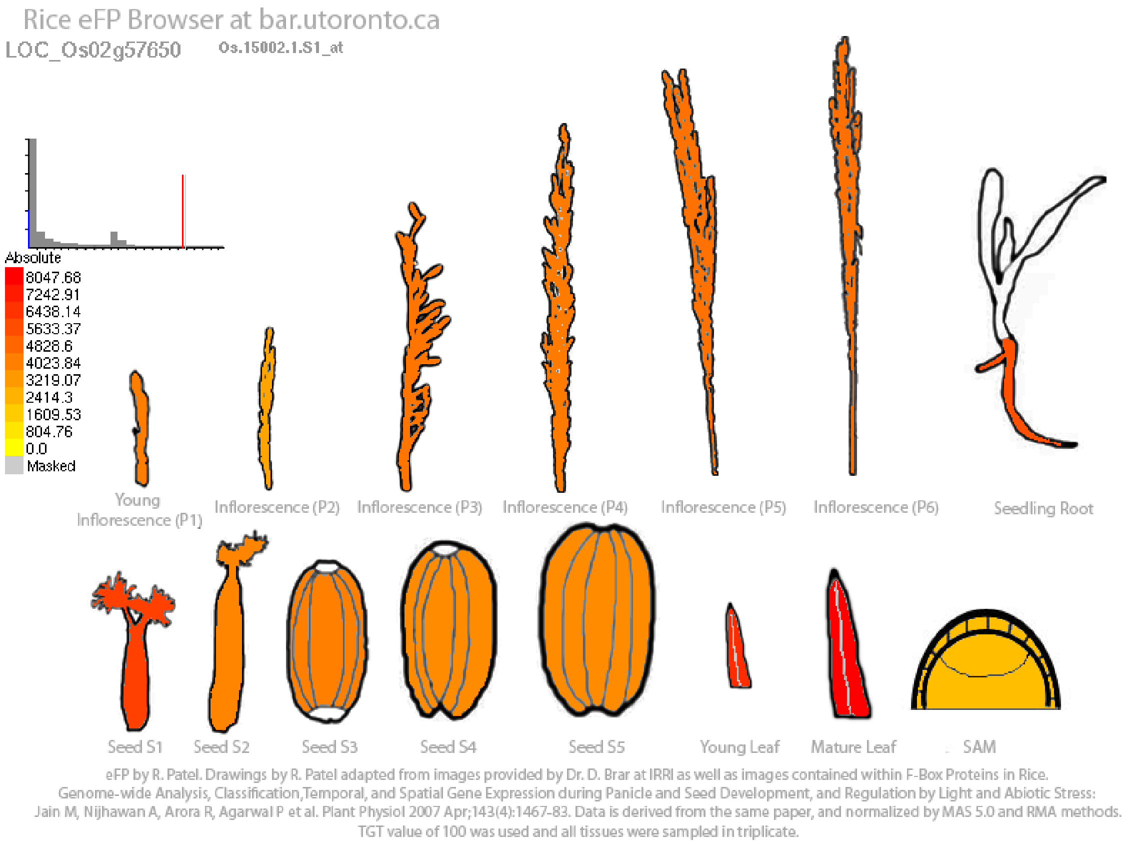

Supplemental Figure 7 | Expression patterns of *OsNTL6*. Relative expression levels of OsNTL6 in inflorescences, seedlings, seeds, young leaves, mature leaves and SAM based on microarray data showed in the eFP browser (http://www.bar.utoronto.ca/efp/cgi-bin/efpWeb.cgi). The color scale shows microarray signal level.
